# Editorial: Task-Related Brain Systems Revealed by Human Imaging Experiments

**DOI:** 10.3389/fnbeh.2022.889486

**Published:** 2022-04-19

**Authors:** Yuji Naya, Kuniyoshi L. Sakai

**Affiliations:** ^1^School of Psychological and Cognitive Sciences, Peking University, Beijing, China; ^2^IDG/McGovern Institute for Brain Research at Peking University, Beijing, China; ^3^Beijing Key Laboratory of Behavior and Mental Health, Peking University, Beijing, China; ^4^Department of Basic Science, Graduate School of Arts and Sciences, The University of Tokyo, Tokyo, Japan

**Keywords:** neuroimaging (functional), human, non-human primates, task design, brain systems, language, cognition

More than three decades have passed since the development of functional magnetic resonance imaging (fMRI), a non-invasive neuroimaging technique that allows us to look into neural activity of the human brain measured by local blood oxygenation level-dependent (BOLD) signals (Ogawa et al., 1990). Progress in neuroimaging studies has clarified a number of brain systems that are critical in higher cognitive functions, including learning and memory. Although “default mode networks” have been assessed without using any tasks, task-design development is still vitally important to reveal the specific brain networks responsible for individual cognitive functions. Therefore, in this Research Topic our goal was to address how task designs for cognitive neuroscience can be advanced, and which specific questions about cognitive functions can be addressed by neuroimaging approaches.

Considering the accumulation of tasks reported in previous neuroimaging and psychological studies, it would be a good start to utilize well-established tasks (e.g., a delayed matching-to-sample task) and combine those tasks with new stimuli and/or stimulus presentation conditions. For example, Zhou et al. used a “dual-feature delayed matching task” to examine the neural mechanisms underlying an attentional function. In their task, the participants attended to either the color or shape of stimuli, and the trans-magnetic stimulation to the right posterior superior temporal sulcus facilitated feature discrimination. In another study, Tsuruha and Tsukiura used a word-face association memory task, where face stimuli were categorized into two age-groups. They examined the effects of in-group (participants with ages close to those of the stimuli) and out-group members (participants with ages different from those of the stimuli) on the neural mechanisms underlying the recollection of association memory. Regarding stimulus presentation conditions, Chen and Naya took over a series of studies (Chen and Naya, 2020a,b) to examine a scene perception using a delayed matching task, in which the identity of an object and its location were encoded under two conditions: a foveal-view (F-V) and a peripheral-view (P-V). Under the F-V condition, the location information of an object was obtained as a gaze position, while under the P-V condition, that information was obtained as a peripheral retinotopic position. In an electrophysiological study of non-human primates, they found robust location signals in the ventral visual pathway, as well as an integration of object and location information in the medial temporal lobe only under the F-V condition.

It is worth noting that task conditions can change significantly according to the status or training of participants. Khaksari et al. used a motor task under either a self-action (actor) or an observation (observer) condition, and observed brain lateralization when participants were actors. On the other hand, Ohbayashi conducted a series of studies to examine motor learning in non-human primates using two sequential reaching tasks (one visually-guided [random] and the other memory-guided [repeating]), while, respectively, inactivating the corresponding motor areas (Ohbayashi et al., 2016; Ohbayashi, 2020). In a subsequent article, Ohbayashi discussed the distinct roles of the motor areas, especially the dorsal premotor cortex and primary motor cortex, on the effects of training over 100 daily sessions. Although such repetitive and intensive training is usually difficult to study in human participants, Sakai et al. focused on second language acquisition in visitors to Japan over the course of several *months*, and succeeded in revealing functional changes in both modality-dependent networks and domain-special language areas. Moreover, these cortical regions were found to be selectively recruited for specified music processes (pitch, tempo, stress, and articulation) after several *years* of musical instrument training (Sakai et al., 2021).

To investigate neurological symptoms, some psychological tasks designed for patients have been tested in non-human primates as an animal model. Misonou and Jimura reversed this common procedure, and tested decision making processes (an immediate small reward vs. a delayed large reward) in human participants given a liquid supply like that used in monkey experiments. Another direction for a new task paradigm would be the use of multi-voxel pattern analyses (MVPA), with which residual bottom-up or top-down signals can be subtracted out from the original signals in each brain region. Pham et al. compared a visual perception task and a visual imagery task, which involved more salient bottom-up and top-down signals, respectively. Yuen et al. examined attentional effects on brain activity during a driving simulation task, suggesting the importance of oculomotor behavior.

It would be also interesting to conduct human neuroimaging studies in which our daily lives are represented or simulated using virtual-reality techniques or natural methods that avoid artificial rule learning. Umejima et al. examined the effects of the use of either paper notebooks or mobile devices on a subsequent memory recall, and found enhanced activations in the hippocampus, visual cortices, and language-related frontal regions for the group using paper notebooks. Moreover, during the natural acquisition of a new language, activations in the bilateral frontal/temporal regions were maintained at a higher level than the initial level during subsequent new grammar conditions for multilinguals (Umejima et al., 2021). These results suggest that individual brain networks become increasingly specialized and intricate to adapt to a constantly changing outer world.

Overall, the above-mentioned findings indicate that hypothesis-driven or top-down approaches are crucial in cognitive or systems neuroscience, together with insights into experimental design. This is why a sophisticated task is required for human neuroimaging studies, especially when studying functions such as cognition, thinking, and language. Such sophistication of task would also be crucial for electrophysiological/imaging studies of non-human primates, which contribute to our understanding of basic brain functions. The above-mentioned findings also open up new and attractive questions about human mind, which could be addressed in future research with much improved and sophisticated task designs.

## Author Contributions

Both authors listed have made a substantial, direct, and intellectual contribution to the work and approved it for publication.

## Conflict of Interest

The authors declare that the research was conducted in the absence of any commercial or financial relationships that could be construed as a potential conflict of interest.

## Publisher's Note

All claims expressed in this article are solely those of the authors and do not necessarily represent those of their affiliated organizations, or those of the publisher, the editors and the reviewers. Any product that may be evaluated in this article, or claim that may be made by its manufacturer, is not guaranteed or endorsed by the publisher.

## References

[B1] Chen H. Naya Y. (2020a). Forward processing of object-location association from the ventral stream to medial temporal lobe in nonhuman primates. Cereb. Cortex 30, 1260–1271. 10.1093/cercor/bhz164 31408097

[B2] Chen H. Naya Y. (2020b). Automatic encoding of a view-centered background image in the macaque temporal lobe. Cereb. Cortex. 30, 6270–6283. 10.1101/2020.03.01.971507 32637986

[B3] Ogawa S. Lee T. M. Kay A. R. Tank D. W. (1990). Brain magnetic resonance imaging with contrast dependent on blood oxygenation. Proc. Natl. Acad. Sci. U.S.A. 87, 9868–9872. 10.1073/pnas.87.24.9868 2124706 PMC55275

[B4] Ohbayashi M (2020). Inhibition of protein synthesis in M1 of monkeys disrupts performance of sequential movements guided by memory. eLife 9, e53038. 10.7554/eLife.53038 32039760 PMC7010406

[B5] Ohbayashi M. Picard N. Strick P. L. (2016). Inactivation of the dorsal premotor area disrupts internally generated, but not visually guided, sequential movements. J. Neurosci. 36, 1971–1976. 10.1523/JNEUROSCI.2356-15 26865620 PMC4748079

[B6] Sakai K. L. Oshiba Y. Horisawa R. Miyamae T. Hayano R. (2021). Music-experience-related and musical-error-dependent activations in the brain. Cereb. Cortex. 10.1093/cercor/bhab478 [Epub ahead of print]. PMC9528789 34937087

[B7] Umejima K. Flynn S. Sakai K. L. (2021). Enhanced activations in syntax-related regions for multilinguals while acquiring a new language. Sci. Rep. 11, 7296. 10.1038/s41598-021-86710-4 33790362 PMC8012711

